# Exhaled Nitric Oxide and Exhaled Breath Temperature as Potential Biomarkers in Patients with Pulmonary Hypertension

**DOI:** 10.1155/2018/7292045

**Published:** 2018-08-26

**Authors:** Giovanna Elisiana Carpagnano, Alessandro Radaeli, Donato Lacedonia, Michele Correale, Giuseppe Carpagnano, Antonio Palmiotti, Maria Pia Foschino Barbaro, Matteo Di Biase, Natale Brunetti, Giulia Scioscia, Mario Malerba

**Affiliations:** ^1^Sezione di Malattie dell'Apparato Respiratorio, Dipartimento di Scienze Mediche e Chirurgiche, Universita' di Foggia, Foggia, Italy; ^2^Dipartimento di Emergenza Urgenza, Spedali Civili di Brescia, Brescia, Italy; ^3^Cardiologia Universitaria, Dipartimento di Scienze Mediche e Chirurgiche, Universita' di Foggia, Foggia, Italy; ^4^Malattie dell'Apparato Respiratorio, Dipartimento di Medicina Traslazionale, Università del Piemonte Orientale, Novara, Italy

## Abstract

**Background:**

Pulmonary hypertension (PH) is a progressive fatal disease thus, noninvasive prognostic tools are needed to follow these patients. The aim of our study was to evaluate fractional exhaled nitric oxide (FeNO) and exhaled breath temperature (EBT) values in patients with PH from different causes and to correlate them with respiratory functional data.

**Methods:**

Twenty-four PH patients underwent spirometry, carbon monoxide diffusion (DLCO) test, transthoracic echocardiography, right-heart catheterization, and FeNO and EBT measurements.

**Results:**

We studied 3 groups according to the type of PH: 10 patients with pulmonary arterial hypertension (PAH) (group A), 11 patients with PH due to chronic obstructive pulmonary disease (COPD) (group B), and 3 patients with PH associated with left heart disease (group C). Mean FeNO values tend to be higher in group B (15.0 ± 9.3ppb) compared with other groups (respectively, 9.9 ± 5.7 and 8.5 ± 5.2 ppb in groups A and C; p = 0.271) but no statistical significance has been reached. Mean values of alveolar NO concentration (CANO) were higher in groups A and B compared to group C (respectively, 16.9 ± 12.6; 13.9 ± 6.8; and 6.7 ± 2.0 ppb) (p = 0.045). EBT mean values were significantly lower in group C when compared with other groups (group C: 29.0 +- 1.3°C, groups A and B: 30.9 ± 1.3 and 31.2 ± 1.2°C, respectively: p = 0.041). EBT levels were inversely correlated to mean pulmonary artery pressure (PAPm) levels (Spearman coefficient -0.481; p = 0.017).

**Conclusions:**

eNO, CANO, and EBT have been evaluated in three groups of PH patients. Interestingly EBT reduction was correlated with PAPm increase, whereas FeNO was higher in COPD patients and CANO in PAH and COPD groups. Further studies are needed to clarify EBT, FeNO, and CANO roles as biomarkers in the monitoring of patients with PH.

## 1. Background

Pulmonary hypertension (PH) is a disorder associated with multiple clinical conditions; it is characterized by increased vascular resistance and pressure overload of the right ventricle. PH can be correlated to the majority of cardiovascular and respiratory disorders in different stages of these diseases. PH is defined as an increased mean pulmonary arterial pressure (PAPm) ≥25 mmHg at rest as assessed by right-heart catheterization (RHC) [1]. The mechanism leading to PH is an imbalance between vasoconstrictors agents and vasodilators such as nitric oxide (NO) in pulmonary circulation [2]. PH is a progressive fatal disease; thus, easy usable and noninvasive prognostic tools are needed for the management of the follow-up of these patients. NO is a strong vasodilator agent and it is synthesized from L-arginine by NO synthase located in different cells of the respiratory tract [3]. A part of NO exhaled during breath flows throughout airways and it is detectable in the expired air as fractional exhaled NO (FeNO). FeNO is usually used as a noninvasive marker of lung inflammation in asthma and other pulmonary diseases since it has been observed that its concentration in the exhaled air is related to the presence of eosinophilic infiltration of the airways [4]. FeNO in patients with PH has been evaluated in a limited number of studies [5–10] with very different results. The alveolar contribution to the exhaled NO (CANO) can be estimated by an extended NO analysis [11]. This has been widely applied in a variety of lung diseases and also in patients with PH founding increased alveolar NO concentrations probably reflecting dysfunctional alveolocapillary diffusion [12]. Other biomarkers in the exhaled air have been determined and among them the exhaled breath temperature (EBT) achieved great interest [13]. Whereas mucosal blood flow is the major contributor to airway temperature, EBT has been used as a marker of inflammation and airway vascularization especially in patients with asthma [14] and COPD [15]. Although the clinical use of this parameter is still uncertain, this measurement is now validated and clear reference limits in healthy subjects have been determined [16].

The aim of this study is to describe FeNO, EBT, and functional test data of the study population. The secondary endpoint is to look for a correlation between FeNO, CANO, and EBT values and pulmonary and cardiac functional data in a group of patients affected by different causes of PH.

## 2. Patients and Methods

### 2.1. Patients

We conducted a cross-sectional, observational study including patients with PH in follow-up at the Cardiac Unit, University Hospital of Foggia. All participants voluntarily signed the informed consent form. The study was conducted in accordance with the principles of the Helsinki Declaration and was approved by Local Ethic Institutional Board (Approval Number 17/CE/2014).

Demographics, comorbid diseases, PH type, World Health Organization (WHO) functional class status, and right-heart catheterization findings assessed at the time of diagnosis have been recorded. All patients underwent pulmonary function tests, carbon monoxide diffusion (DLCO) test, six-minute walking distance determination, transthoracic echocardiography, and FeNO and EBT levels determination. Exclusion criteria were smoking within the preceding 6 months, respiratory tract infection within the preceding 2 weeks, history of hyperreactive airways disease, pregnancy, corticosteroid use with equivalent dose >10 mg of prednisone daily, and use of L-arginine, nitrates, or phosphodiesterase inhibitors.

### 2.2. Pulmonary Function Tests

Static and dynamic lung volumes were measured using the nitrogen washout method and a pneumotachograph with volume integrator (CAD/Net system 1070; Medical Graphics Corporation; St. Paul, MN) in accordance with the criteria of the American Thoracic Society (ATS) [17]. Lung carbon monoxide diffusion capacity (DLCO) was assessed by means of the single-breath method (PF/DX system; Medical Graphics Corporation) with the patients in the sitting position [18]. The indexes were expressed as percentages of the predicted normal.

### 2.3. Six Minutes Walking Distance

Walking capacity was evaluated by means of the distance covered during a six-minute walking test (6MWD) according to the ATS statement [19]

### 2.4. Echocardiography

Conventional echocardiography was used to assess left ventricular dimensions and ejection fraction, peak velocities of trans-mitral early (E) and late diastolic (A) LV filling, the ratio of trans-mitral early to late (E/A ratio) left ventricular filling velocity, and E-deceleration time.

Pulmonary artery systolic pressures were estimated using the approach of calculating the systolic pressure gradient between right ventricle and right atrium by the maximum velocity of the tricuspid regurgitant jet, using the modified Bernoulli equation, and then adding to this value the estimated right atrial pressures based on both the size of the inferior vena cava and the change in calibre of this vessel with respiration, according to international recommendations [20].

Transthoracic echocardiography was performed with the use of iE33 (Philips Medical Systems, Andover, MA, USA). All echocardiographic studies were performed and interpreted by experienced physicians. They were blinded of the clinical data.

### 2.5. Right-Heart Catheterization

Hemodynamic assessment was performed by right-heart catheterization (RHC), according to recent guidelines [1]. Pulmonary arterial (systolic, diastolic, and mean), right atrial, and pulmonary artery wedge pressures (PAWP) were recorded at the end of a quiet respiratory cycle. Oxygen saturations in the superior vena cava, inferior vena cava, pulmonary artery, and femoral artery were obtained. Pulmonary vein saturation was assumed at 98%. Pulmonary and systemic flows were obtained by the Fick principle using table-derived oxygen consumption values and calculated oxygen content at the correspondent different sites. Pulmonary and systemic vascular resistance indices were calculated using the standard formula.

### 2.6. Exhaled Breath Temperature Measurement

EBT was measured with X-halo device (Delmedica Investments, Singapore) according to previously validated methods [13]. Briefly, patients were requested to inhale freely through the nose and then to exhale into the device at a rate and depth typical of their normal tidal-breathing rhythm. Ambient temperature was tested and recorded for each measurement with an external thermometer. We measured EBT to different ambient temperature that ranged from 0 to 32°C.

### 2.7. Measurement of Fractional Exhaled NO

Measurement of FeNO was performed according to recent guidelines (American Thoracic Society) [4, 21]. The Medisoft FeNO+ device, which is a semiportable for repeatable multiflow measurement of exhaled NO with offline measurement, was used. It has a software package that provides step-by-step online quality control. The measurement range is 0–600 ppb [22]. FeNO was measured using a previously described restricted breath technique, which employed expiratory resistance and positive mouth pressure to close the velum and exclude nasal NO: expiratory flow measurements at 50 mL/s, 100 mL/s, and a 350 mL/s have been evaluated. Repeated exhalations were performed until three plateaus agreed within 5% of interobservation difference. All the enrolled patients were able to complete the measurements. The contributions of the bronchi (bronchial NO flux) and the alveoli (alveolar concentration of NO: CANO) to FeNO will be derived from regression analysis, with NO output as the dependent and exhalation flow rate as the independent factor. The slope and intercept of the regression line are approximate values of alveolar NO concentration and bronchial NO flux, respectively [23].

### 2.8. Statistical Analysis

Statistical analysis has been performed using SPSS 21.0 software (SPSS 21.0 for Windows; SPSS, Chicago, Illinois). Descriptive statistics were reported as mean, standard deviation (SD), median, and minimum-maximum. Categorical variables were expressed as case numbers and percentages. The normality of distribution of variables was examined using Kolmogorov-Smirnov and Shapiro-Wilk tests. Comparison between two variables was assessed using the paired t-test or Wilcoxon test where appropriated. Comparisons among more than two variables were performed by ANOVA or Kruskal-Wallis test as appropriate. Comparisons of categorical variables were made using the square *χ* test. Spearman correlation test was used to examine the relationship between the variables. A p<0.05 was considered as statistical significant.

## 3. Results

Demographic characteristics of the studied patients are reported in Table 1. In total 24 patients were enrolled. Patients were divided into 3 groups according to the classification of PH adopted by European Society of Cardiology guidelines [1]: group A was composed of 10 patients with pulmonary arterial hypertension (PAH) (3 patients with idiopathic PH, 6 patients with PH secondary to connective tissue disease, and 1 patient with portal hypertension). Group B was composed of 11 patients with PH secondary to chronic obstructive pulmonary disease (COPD) (in accordance with GOLD recommendations) [24]. Finally, data about 3 patients with heart failure (HF) have been analysed as a separate group (group C: PH associated with HF). Smoking history was present in 5 patients (19.2%) (average 20.1 pack-years) and all patients quit smoking before the study as established by inclusion criteria of the study.

The whole cohort of patients had a moderate to severe WHO functional class: five patients were in class II, 17 in class III, and two in class IV. Patients were mostly men (69.2%) with normal BMI (patients in the group C showed a lower BMI compared to other groups as reported in Table 1). Four patients showed chronic respiratory failure (all of them included in group B). No patients had a documented interstitial lung disease. The prevalences of comorbid diseases are reported in Table 1.

The PAPm was 35.5 ± 8.3 mmHg, mean pulmonary capillary wedge pressure (PAWP) was 13.0 ± 4.6 mmHg, and pulmonary vascular resistance was 4.7 ± 2.2 wood units (WU) at right-heart catheterization. PAPm was higher in group C compared with the other two groups (data reported in Table 2). Patients with PH associated with respiratory disease (COPD) had lower mean values of Tiffeneau index (FEV_1_/FVC) compared with other groups (bronchial obstruction pattern at pulmonary function tests). Patients with PAH and with PH associated with COPD (groups A and B) showed impaired DLCO (<70% predicted). Right-heart catheterization and respiratory function data are represented in Table 2. FeNO measurements have been completed by all patients. Mean FeNO50 values were 11.9 ± 7.6 ppb in the study population. These data conform to what is generally considered the normal limits of FeNO (10.8 ± 47ppb) [25]. Mean CANO and EBT values were 14.1 ± 9.7 ppb and 30.7 ± 1-4°C, respectively.

**Figure 1:**
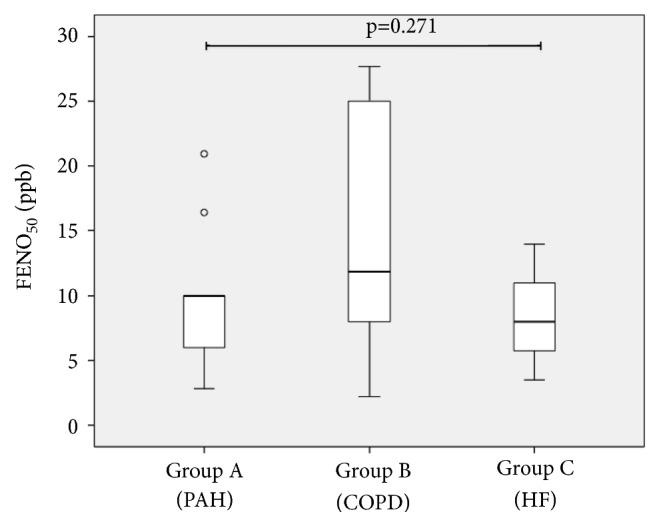
Boxplot showing FENO_50_ (fractional exhaled nitric oxide at 50 ml/sec flow) values in the studied groups. Thick line showing median, box showing 25% and 75% interquartile ranges, and error bars showing minimum and maximum values. Small circles indicate outlier values. Groups were compared by ANOVA (PAH, pulmonary artery hypertension; COPD, chronic obstructive pulmonary disease; and HF, heart failure).

We recorded some interesting differences among the different PH groups in the comparison between FeNO and EBT values (results are reported in Table 3). Mean FeNO values were higher in group B: 15.0 ± 9.3 ppb compared with group 1 and group 3 (respectively, 9.9 ± 5.7 and 8.5 ± 5.2 ppb) even if this trend was not statistically significant (p = 0.271, Figure 1).

**Figure 2:**
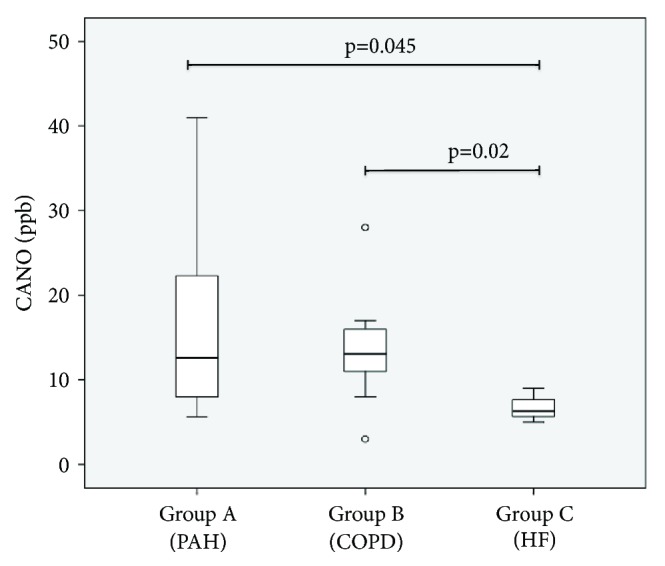
Boxplot showing CANO (alveolar concentration of nitric oxide) values in the studied groups. Thick line showing median, box showing 25% and 75% interquartile ranges, and error bars showing minimum and maximum values. Small circles indicate outlier values. Groups were compared by ANOVA and Wilcoxon test (PAH, pulmonary artery hypertension; COPD, chronic obstructive pulmonary disease; and HF, heart failure).

**Figure 3:**
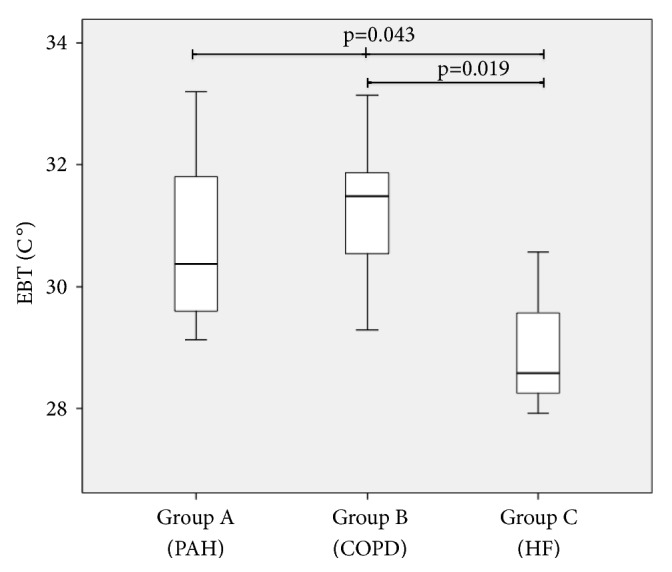
Boxplot showing EBT (exhaled breath temperature) values in the studied groups. Thick line showing median, box showing 25% and 75% interquartile ranges, and error bars showing minimum and maximum values. Groups were compared by ANOVA and Wilcoxon test (PAH, pulmonary artery hypertension; COPD, chronic obstructive pulmonary disease; and HF, heart failure).

**Figure 4:**
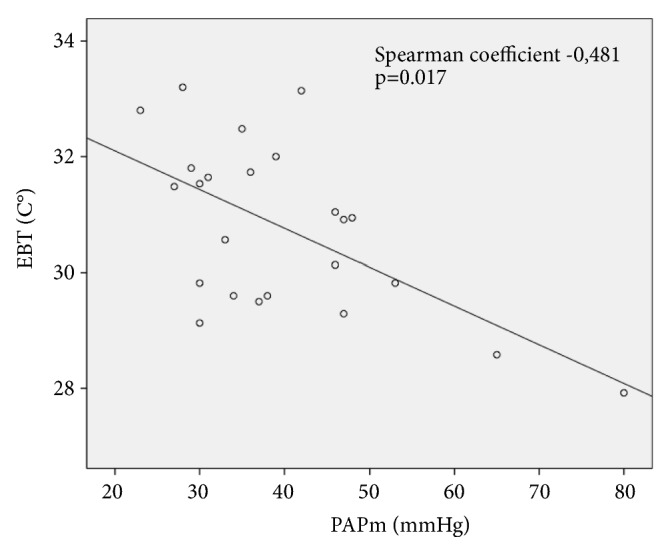
Scatterplot showing correlation between EBT (exhaled breath temperature) and PAPm (mean pulmonary arterial pressure) in the studied patients (whole group).

Mean values of CANO were higher in groups A and B (16.9 ± 12.6; and 13.9 ± 6.8 ppb) compared with the values observed in group C (6.7 ± 2.0 ppb) (p = 0.045 Figure 2). We also found lower mean EBT values in the group C (PH associated with HF) if compared with groups A and B ones (30.9 ± 1.3°C versus 30.6 ± 1.3 and 31.2 ± 1.2°C, respectively, p = 0.041 Figure 3). EBT levels were inversely correlated to PAPm levels, with a Spearman coefficient of -0.481; p = 0.0217 (Figure 4). On the other hand, FeNO and CANO levels showed no correlation with any other variable analysed or with comorbidities in the patients studied groups.

## 4. Discussion

We conducted a cross-sectional, observational study on a group of patients with PH, whose diagnosis was confirmed by RHC, evaluating pulmonary function tests and FeNO and EBT determination. This is one of the few studies directly assessing RHC data and correlating with the respiratory functional ones.

The main findings of this study were as follows: (i) FeNO values were different between groups, even though nonstatistically significant, with higher values observed in respiratory group (group B) showing the presence of enhanced NO from the large airways; (ii) CANO levels were higher in PAH and respiratory groups (groups A and 2) probably reflecting the reduced diffusion capacity from alveolar to vascular space as demonstrated by the impaired DLCO observed in these patients; and (iii) EBT was lower in group C showing an inverse relationship with PAPm values.

The role of endogenous production of NO in PH up to now is still uncertain and very controversial. Previous published data demonstrated that FeNO levels are reduced or normal in patients with PH, even considering the different classification groups of patients; [7, 26–28] some authors found that FeNO levels are especially elevated when associated with systemic sclerosis and interstitial lung diseases (ILD) [29]. In summary, available literature data about FeNO values in patients with PH describe reduced levels if compared with normal subjects except in cases of airways or alveolar inflammation. The underlying mechanisms associated with these reduced levels of FeNO in PH patients may include (I) the reduction of substrate for NO production as demonstrable by the fact that L-arginine oral administration increased NO levels in precapillary PH [30]; (II) reduced NO production by endothelial NO synthase (eNOS), as in idiopathic PAH the levels of dimethylarginine (a potent eNOS inhibitor) are higher [31]; and (III) reduced pulmonary expression of eNOS itself has been hypothesized in PAH too [32]. Finally a reduced diffusion capacity of NO from alveolar to vascular space due to pathological modifications of the pulmonary tissue may support the reduced FeNO values in PH patients. Probably our results are explained by the fact that the underlying respiratory disease in group B induces a certain degree of airway inflammation that supports the (modest) production of bronchial NO, although within the limits of normality [25].

An increase in CANO values has been already observed in previous published papers [7, 9]; it was related to the presence of a preserved alveolar synthesis of NO in presence of a reduced alveolar vascular-diffusion due to tissue pathological alterations. Our results are in line with these data as we observed reduced DLCO levels in case of higher CANO ones. If we consider the previous literature CANO normal levels ranging about 1.8 ± 0.2 ppb [7], the levels found in all the groups of our cohort were higher and this could be probably explained by the fact that all showed decrease DLCO values. It is interesting to highlight that elevated CANO levels were observed in PH patients with involvement in local pulmonary pathophysiology (IPAH, connective disease, COPD, etc.). Our data confirmed that CANO levels were lower in types of PH associated with heart failure.

To our best knowledge, this is the first study analysing EBT in PH patients. Recently EBT normal levels have been established ranging about 30.66 C° measured at 22°C in nonsmoking healthy subjects [16]. According to previous published data we confirmed that EBT was lower in patents with more severe PH (as demonstrated by the inverse relationship with PAPm). These data suggest that reduced EBT values may reflect a decreased bronchial vascularization due to vascular degeneration in more advanced stages of PH, as the matter of the fact that mucosal blood flow is the major contributor to airway temperature. Literature data about EBT underline a possible relationship between asthma inflammation and airways remodelling too [33]. Moreover, in asthma elevated rates of exhaled breath temperature changes and bronchial blood flow have been related to increased vascularity of the airway mucosa as a result of inflammation [34]. In the same way, we may hypothesize that some mechanisms leading to HP alteration of bronchial mucosal blood flow may be revealed by the reduction of EBT when compared to normal values, especially in patients with high levels of PAPm; this may reflect the changes in bronchial blood flow and heat exchange resulting in decreased bronchial vascularization. A comparison of cardiac index values did not show any significant difference among groups suggesting that blood flow alterations should be localized only in bronchial mucosa. According to our data, we observed a direct correlation between 6MWD and EBT (spearman = 0.539 p = 0.021) suggesting that low levels of EBT may predict the clinical severity of PH in these patients

One of the possible limitations of the study is the low number of subjects included, because PH types are numerous and with different underlying diseases due to equally different pathophysiological mechanisms. Therefore, the study of homogenous group of patients with PH is also difficult in relation to the relative rarity of the disease. In particular, the small number of patients with HF enrolled in group 3 compels us to be extremely cautious in generalizing the results of the study. Another possible limitation could be the lack of data about airway inflammatory cell patterns of the studied patients. These limitations make difficult to establish whether patients enrolled with COPD could be classified as overlap of asthma and COPD (ACO); however, patients in group B had no history of atopy or asthma or bronchial reversibility exceeding 12% or 200 ml (which are major criteria for the diagnosis of ACO) [35]

## 5. Conclusions

In summary our data show that the different biomarkers evaluated in different groups of patients with PH could be in relation to the pathophysiological nature of the disease. The most original and interesting result is the association between EBT reduction and PAPm increase. This relationship could be due to an alteration of bronchial blood flow and reflects the presence of a “remodelling” of airways structures in these patients. These data, if confirmed by larger trials, open new perspectives for future studies aimed at assessing a possible role of FeNO, CANO, and particularly EBT as biomarkers in PH and in the clinical monitoring of these patients.

## Figures and Tables

**Table 1:** Demographic characteristics of patients studied.

	All	Group A (PAH)	Group B (COPD)	Group C (HF)	p
number of subjects	24	10	11	3	
Age, years	63.2 ± 12.2	65 (11.2)	64.1 (14.4)	62.3 (10.5)	0.950
Male, n (%)	16 (66.6)	6 (60)	9 (81)	1 (33.3)	0.242
Bmi Kg/m^2^	27.2 ± 4.3	27.7 (5.1)	28.1 (3.4)	23.2 (1.7)	0.229
Smoking history, n (%)	5 (19.2)	0	5 (45.4)	0	<0.01
Pack-years	20.1		20.1		
WHO functional class, n (%)					
Class II	5 (20.8)	2 (20)	2 (18)	1 (33.3)	0.888
Class III	17 (70.8)	8 (80)	7 (63.6)	2 (66.6)	
Class IV	2 (8.4)	0	2 (18)	0	
Co-morbid diseases, n (%)					
Systemic Art. Hypertension	7 (26.9)	5 (50)	1 (9)	1 (33.3)	0.311
Diabetes mellitus	7 (26.9)	3 (30)	2 (18)	1 (33.3)	0.977
Ischemic Cardiac disease	4 (15.3)	1 (10)	1 (9)	2 (66.6)	0.999
Chronic renal failure	5 (19.2)	3 (30)	1 (9)	1 (33.3)	0.726

Data reported as mean ± standard deviation or frequencies.

PAH: pulmonary arterial hypertension; COPD: chronic obstructive pulmonary disease; HF: heart failure; WHO: World Health Organization; Art.: arterial; n.s.: not significant; and p: p value among the groups studied (ANOVA or Chi square)

**Table 2:** Right-heart catheterization and pulmonary function data of patients studied.

	Group A (PHA)	Group B (COPD)	Group C (HF)	p
Mean PAP (mm Hg)	35.5 (8.3)	38.2 (8.3)	59.3 (24)	0.011
	32.5 (28-53)	37 (23-47)	65 (33-80)	
Systolic PAP (mm Hg)	72.7 (21.1)	81.7 (22.3)	101.6 (18.9)	0.158
	70 (48-105)	83 (50-113)	110 (80-115)	
CI (l/min)	2.98 (0.88)	2.89 (0.79)	3.33 (0.57)	0.710
	2.98 (2-5)	2.8 (1-4)	3 (3-4)	
Pulmonary capillary wedge pressure (mm Hg)	13 (4.6)	14.5 (4.4)	16 (1.4)	0.598
	13 (8-22)	14 (10-22)	15 (17-21)	
Pulmonary vascular resistance (WU)	4.7 (2.2)	5.1(3.8)	4.8 (1.8)	0.478
	5.7 (1.8-8.3)	3.6(1.7-11.3)	5 (1.8-10)	
Right atrial pressure (mm Hg)	8.5 (3.6)	10.2 (2.1)	9.5 (2.2)	0.256
	9 (2-13)	9 (8-13)	9 (5-16)	
FEV1 (L)	1.9 (8.85)	1.5 (0.43)	1.13 (0.27)	0.197
	1.7 (1-3.5)	1.5 (0.79-1.9)	1 (0.9-1.43)	
FEV1 (%predicted)	76.7 (17.2)	58.5 (15.9)	57.3 (4.5)	0.071
	81 (55-105)	65 (31-74)	57 (53-62)	
FVC (L)	2.55 (1.1)	2.9 (1)	1.4 (045)	0.158
	2.4 (1.1-4.8)	2.8 (1.3-4.4)	1.3 (1-1.9)	
FVC (% predicted)	82.5 (21.5)	82.8 (19.4)	59.6 (7.6)	0.195
	85 (50-118)	76 (57-120)	58 (53-68)	
FEV1/FVC	74.3 (12.4)	54.6 (11.8)	79.6 (16)	0.007
	73 (53-93)	54 (35-71)	73 (68-98)	
DLCO (%predicted)	37.7 (12.4)	41.4 (13.6)	71.6 (7.6)	0.003
	36 (24-59)	44 (23-63)	70 (65-80)	
6MWD (meters)	336 (140.3)	355.3 (153)	278.3 (158.5)	0.881
	299(160-497)	391(100-520)	207(100-520)	

Data reported as mean (standard deviation) and median (minimum-maximum).

PAP: pulmonary artery pressure; CI: cardiac index; WU: wood units; FEV1: forced expiratory volume in 1 second; FVC: forced vital capacity; DLCO: carbon monoxide diffusion; 6MWD: six-minute walking distance; n.s.: not significant; and p: p value among the groups studied (ANOVA)

**Table 3:** FENO, CANO, and EBT data of all patients with pulmonary hypertension and compared among groups (ANOVA).

		Mean(SD) Median (min-max)		
		All patients		

FENO50 ppb		11.9 (7.6) 10 (2.2-27.6)		
CANO ppb		14.1 (9.7) 12.6 (3-41)		
EBT °C		30.7 (1.4) 30.9 (27.9 – 33.2)		

	Group A (PAH)	Group B (COPD)	Group C (HF)	p

FENO50	9.9 (5.7)	15.0 (9.3)	8.5 (5.2)	
	10 (2.8-20.9)	11.8 (2.2-27.6)	8.0 (3.5-14)	0.271
CANO	16.9 (12.6)	13.9 (6.8)	6.7 (2.0)	
	12.6 (6-41)	13.0 (3-28)	6.3 (5-9)	<0.045
EBT	30.9 (1.3)	31.2 (1.2)	29.0 (1.3)	<0.041
	30.9 (29.1-33.2)	31.4 (29.2-33.1)	28.5 (27.9-30.5)	

Data reported as mean (standard deviation) and median (minimum-maximum).

FENO50: fractional exhaled nitric oxide at expiratory flow 50 ml/sec; CANO: alveolar concentration of nitric oxide; EBT: exhaled breath temperature; PAH: pulmonary arterial hypertension; COPD: chronic obstructive pulmonary disease; n.s.: not significant; and p: p value among the groups studied (ANOVA).

## Data Availability

The data used to support the findings of this study are available from the corresponding author upon request.

## Abbreviations

6MWD: Six-minute walking distance
ACO: Asthma COPD overlap
BMI: Body mass index
COPD: Chronic obstructive pulmonary disease
CANO: Nitric oxide alveolar concentration
DLCO: Carbon monoxide diffusion
EBT: Exhaled breath temperature
FeNO: Fractional exhaled nitric oxide
FEV1: Forced expiratory volume in 1 second
FVC: Forced vital capacity
NO: Nitric oxide
PH: Pulmonary hypertension
PAH: Pulmonary artery hypertension
PAP: Pulmonary artery pressure
PAPm: Mean pulmonary artery pressure
PAWP: Pulmonary artery wedge pressure
RHC: Right-heart catheterization
SD: Standard deviation
WHO: World health organization
WU: Wood units.
